# Sebaceous Carcinoma of the Wrist in an Elderly Woman: A Case Report

**DOI:** 10.7759/cureus.45057

**Published:** 2023-09-11

**Authors:** Akhila M Reddy, Jeremy Purser, Bailey Nelson, Brent Paulger, Cloyce Stetson

**Affiliations:** 1 Department of Dermatology, Texas Tech University Health Sciences Center, Lubbock, USA

**Keywords:** sebaceous tumors, sebaceous neoplasm, ­skin cancer, sebaceous cell carcinoma, extraocular sebaceous carcinoma

## Abstract

Sebaceous carcinoma is a rare, aggressive cutaneous malignancy most commonly arising from the periocular area. Extraocular locations of sebaceous carcinomas, particularly outside of the head and neck region, are rare and not well-described. We report a case of an 89-year-old Caucasian female with sebaceous carcinoma of the right wrist. She initially presented with a 1.2-centimeter friable nodule on the right wrist. Initial shave biopsy and subsequent pathologic evaluation revealed a basaloid neoplasm with sebaceous differentiation, atypia, and frequent mitoses, consistent with sebaceous carcinoma. The presented case reviews common clinical features and the pertinent histopathology of ocular and extraocular sebaceous carcinoma and provides a literature review of diagnosis, prognosis, and treatment.

## Introduction

Sebaceous carcinoma is an aggressive cutaneous malignancy arising from sebaceous glands. It is rare, with an estimated incidence of one to two cases per 1,000,000 person-years [1]. The peak incidence of sebaceous carcinoma occurs from 60-79 years, and it more commonly presents in Caucasian and Asian populations [2,3]. Sebaceous carcinomas most commonly occur in the ocular area and head and neck region, corresponding to the density of sebaceous glands in the skin. Only 5-18% of cases occur elsewhere in the body [2]. Sebaceous carcinoma often presents nonspecifically and can be mistaken for common dermatologic and ophthalmologic conditions [4]. Extraocular locations of sebaceous carcinoma outside of the head and neck region are uncommon and not well-described. Here, we document a unique case of sebaceous carcinoma presenting on the right wrist.

This article was previously presented as a meeting poster at the 35th Texas Tech University Health Sciences Center Graduate School of Biomedical Sciences Student Research Week on March 1, 2023.

## Case presentation

**Figure 1:**
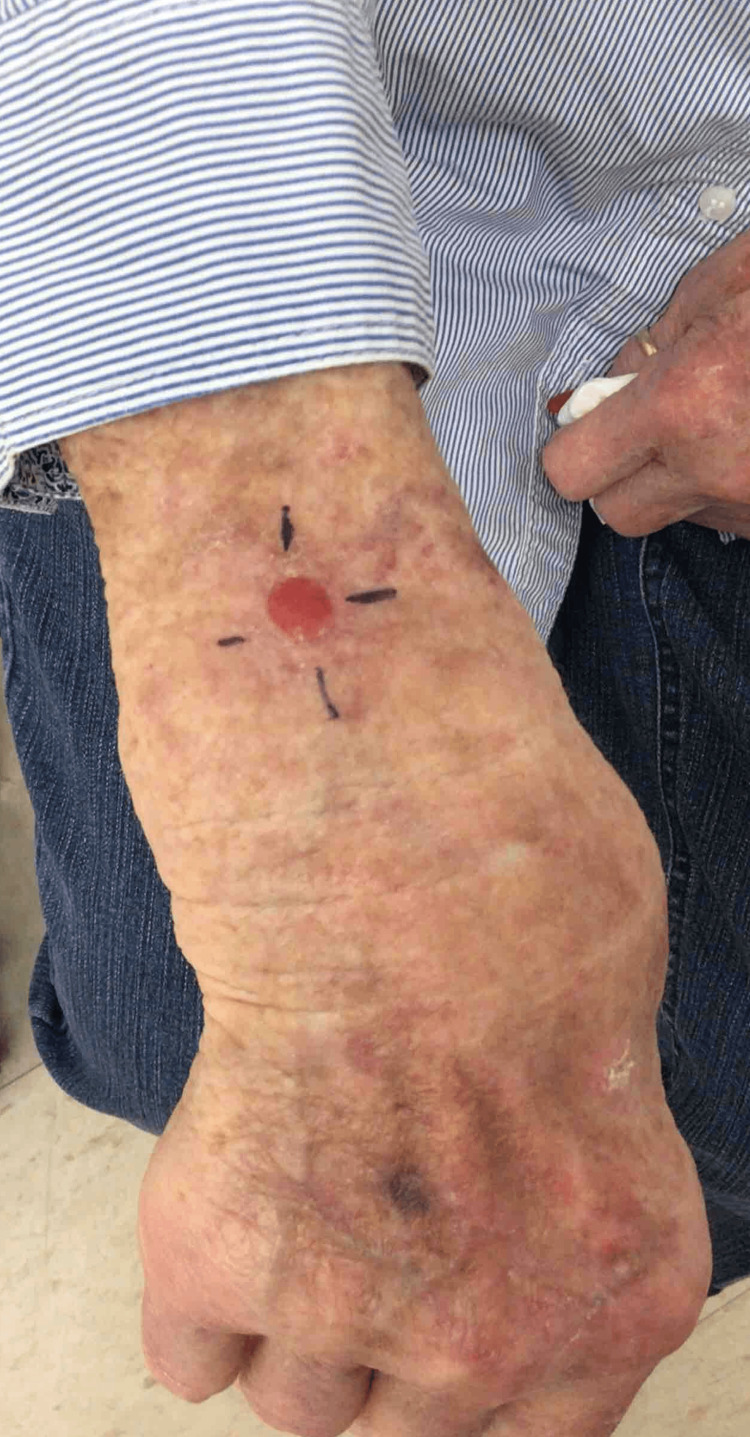
Clinical presentation of 1.2 cm erythematous exophytic lesion on the right dorsal wrist

**Figure 2:**
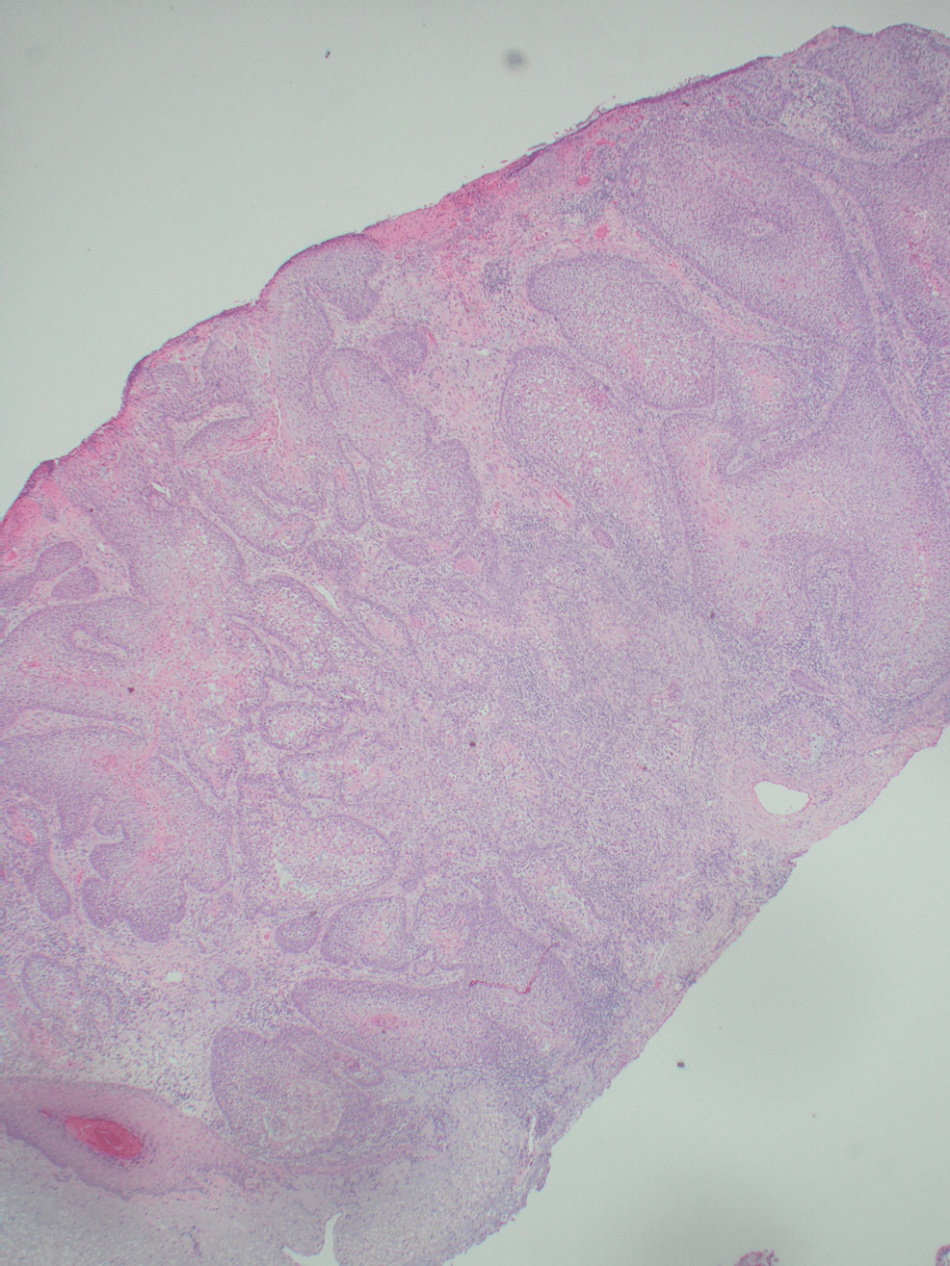
Low-power scan of the shave biopsy showing an infiltrating tumor into the dermis

**Figure 3:**
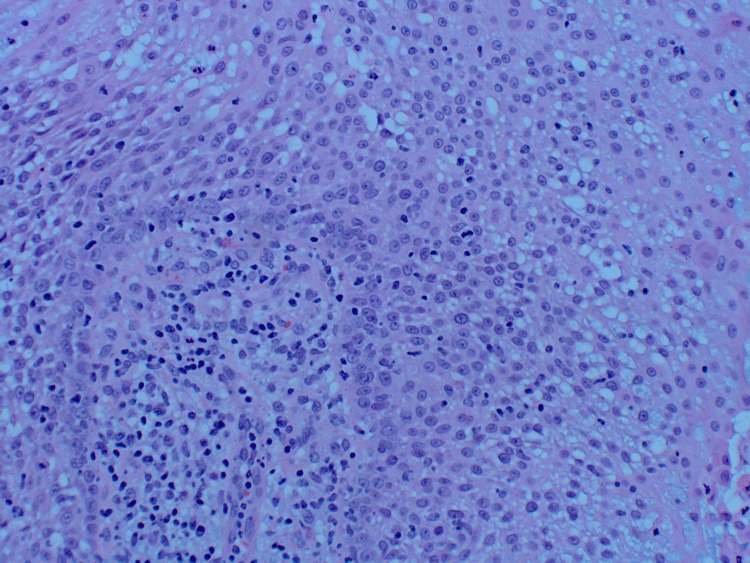
High-power scan of the shave biopsy showing sebaceous differentiation with atypia and numerous mitotic figures

**Figure 4:**
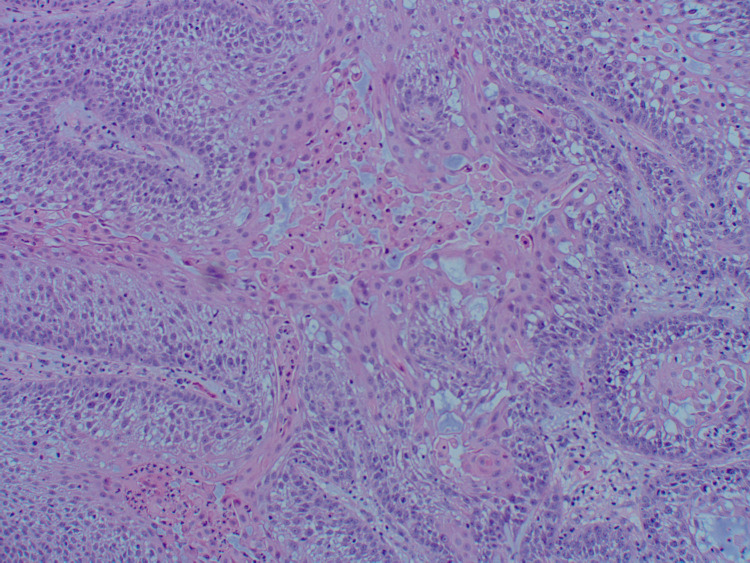
High-power scan of the shave biopsy showing atypical basaloid cells with sebaceous differentiation, consistent with a sebaceous carcinoma

An 89-year-old Caucasian female presented to the dermatology clinic with a 1.2-centimeter, friable, raised, red nodule on the right wrist exhibiting features resembling squamous cell carcinoma (Figure 1). The patient stated the lesion had been present for one month and was stable since onset. No regional lymph node involvement or additional symptoms were reported. Initial shave biopsy of the lesion and subsequent pathologic evaluation revealed a basaloid neoplasm with sebaceous differentiation, moderate atypia, and frequent mitoses (Figures 2-4). The patient also presented with actinic keratosis on the hands, arms, ears, and forehead, and was treated with cryotherapy and prescribed topical 5-fluorouracil/calcipotriene. Her past medical history was significant for melanoma of the nasal tip with negative sentinel lymph node biopsy and melanoma of the left cheek, both surgically excised. Past medical history was also significant for multiple squamous cell carcinomas of the head, also treated with excision. No history of metastasis or visceral malignancy was reported.

## Discussion

Sebaceous carcinoma can be classified as ocular or extraocular depending on its tissue of origin, a distinction that is relevant in presentation, pathology, and treatment [1,2]. Up to 75% of cases of sebaceous carcinoma arise from the ocular adnexa, most often on the upper lid from the Meibominan glands of the tarsus or Zeis glands of the eyelashes [1,2,5]. Extraocular sebaceous carcinoma frequently arises from the skin of the head and neck, corresponding to the density of sebaceous glands [1,2]. Though uncommon, cases like the one we report here have been reported in additional anatomical locations, including the trunk, genitals, extremities, and breast [6-8].

Sebaceous carcinoma typically presents as a nonspecific, painless nodule ranging from pink to yellow in color, though clinical features can vary [9]. Ocular sebaceous carcinoma can also present with diffuse thickening of the eyelid [2]. Ocular sebaceous carcinoma is commonly mistaken for benign conditions such as chalazion, keratoconjunctivitis, and blepharoconjunctivitis, frequently leading to delayed diagnosis with a reported mean delay of 1.0-2.9 years [1,9]. Extraocular sebaceous carcinoma may resemble nonmelanoma skin cancers, including squamous and basal cell carcinoma, as well as benign conditions such as molluscum contagiosum, pyogenic granuloma, or keratoacanthoma [1]. Due to the nonspecific clinical presentation of sebaceous carcinoma, biopsy is essential for diagnosis.

The histological presentation of sebaceous carcinoma varies. The typical presentation is unencapsulated, lobular sebaceous, and undifferentiated cells with characteristic lipid granules present in the cytoplasm of tumor cells. Tumor cells also display classical features of malignancy such as mitotic figures, nuclear hyperchromatism, and nuclear pleomorphism [9]. Sebaceous carcinomas arising from the eyelid are significantly more likely to be poorly differentiated than extraocular sebaceous carcinomas [1]. Positive immunohistochemistry stains for adipophilin, EMA, CK7, Ber-EP4, androgen receptor, and p53 can be utilized in identifying sebaceous carcinoma [10].

The pathology underlying sebaceous carcinoma is unclear and likely involves multiple molecular pathways. P53 and PIK3CA genes are frequently mutated in ocular and extraocular sebaceous carcinomas while NOTCH1 gene mutations occur more frequently in extraocular sebaceous carcinomas alone [11]. Risk factors include immune suppression, radiation therapy, UV exposure, familial retinoblastoma, actinic keratosis, and Muir-Torre syndrome, a clinical variant of hereditary nonpolyposis colorectal cancer [2,12]. Muir-Torre syndrome involves mutations in MLH1, MSH2, and MSH6 DNA mismatch repair genes, and presents with at least one sebaceous gland tumor, commonly extraocular sebaceous carcinoma, along with visceral malignancies, including colorectal, endometrial, ovarian and renal carcinomas. Referral to a geneticist for screening of Muir-Torre syndrome should be considered in patients with sebaceous carcinoma who have a personal history or strong family history of visceral malignancy, or whose tumors demonstrate loss of one or more associated DNA mismatch proteins on immunohistochemistry, if available [9] The patient in the reported case was not screened for Muir-Torre syndrome based on the absence of a history of visceral malignancy and advanced age of presentation. Observed and relative survival rates of sebaceous carcinoma are 78.20% and 92.72%, respectively, at five years and 61.72% and 86.98%, respectively, at 10 years [1]. Extraocular sebaceous carcinoma is more often associated with a better prognosis than ocular sebaceous carcinoma, although existing literature does include conflicting conclusions. Extraocular sebaceous carcinoma has a reduced tendency for metastasis to regional lymph nodes and reduced rates of recurrence relative to ocular sebaceous carcinoma [5,9]. Ocular sebaceous carcinomas can be assessed using the American Joint Committee on Cancer (AJCC) staging system, while extraocular sebaceous carcinomas can be assessed using the Union for International Cancer Control TNM staging system for skin carcinomas [3]. Sentinel lymph node biopsy is not routinely recommended for extraocular sebaceous carcinoma but can be considered for ocular sebaceous carcinoma [3,9].

Treatment modalities for sebaceous carcinoma include wide local excision, Mohs micrographic surgery, surgical excision with complete circumferential peripheral and deep margin assessment (CCPDMA) with frozen or permanent sections, radiotherapy, and chemotherapy with anthracycline-based or platinum-based regimens [2,3]. Evidence-based clinical guidelines suggest margin-controlled therapies, including Mohs micrographic surgery or CCPDMA for the treatment of extraocular sebaceous carcinoma. If CCPDMA or Mohs micrographic surgery is unavailable, wide local excision with margins of 1 centimeter can also be used. Radiotherapy is reserved for surgery-ineligible cases. In the event of nodal involvement, lymph node excision and radiotherapy can be considered. Long-term follow-up should be performed after treatment to monitor for recurrence [3].

## Conclusions

Sebaceous carcinoma is an aggressive cutaneous malignancy that classically presents in the periocular area. Our reported case of sebaceous carcinoma presenting on the wrist illustrates the varied, nonspecific clinical presentation of sebaceous carcinoma. Particularly in extraocular regions, sebaceous carcinomas can resemble nonmelanoma skin cancers, as reported here, along with other benign skin conditions. Providers should not exclude sebaceous carcinoma from their differential based on the anatomical location of the presenting lesion. Suspicious lesions should be biopsied with subsequent histopathologic analysis for a definitive diagnosis of sebaceous carcinoma.
